# Accuracy of Expected Symptoms and Subsequent Quality of Life Measures Among Adults With COPD

**DOI:** 10.1001/jamanetworkopen.2023.44030

**Published:** 2023-11-21

**Authors:** Joanna L. Hart, Amy E. Summer, Lon Ogunduyile, Folasade C. Lapite, David Hong, Casey Whitman, Bryan S. Blette, Michael O. Harhay, Scott D. Halpern

**Affiliations:** 1 Palliative and Advanced Illness Research Center, University of Pennsylvania, Philadelphia; 2 Division of Pulmonary, Allergy, and Critical Care, Department of Medicine, University of Pennsylvania, Philadelphia; 3 Department of Medical Ethics and Health Policy, University of Pennsylvania, Philadelphia; 4 Corporal Michael J. Crescenz VA Medical Center, Philadelphia, Pennsylvania; 5 Tulane University School of Medicine, New Orleans, Louisiana; 6 Department of Biostatistics, Epidemiology and Informatics, University of Pennsylvania, Philadelphia; 7 Department of Biostatistics, Vanderbilt University, Nashville, Tennessee

## Abstract

**Importance:**

Patients’ expectations for future health guide their decisions and enable them to prepare, adapt, and cope. However, little is known about how inaccurate expectations may affect patients’ illness outcomes.

**Objective:**

To assess the association between patients’ expectation inaccuracies and health-related quality of life.

**Design, Setting, and Participants:**

This cohort study of patients with severe chronic obstructive pulmonary disease (COPD) was conducted from 2017 to 2021, which included a 24-month follow-up period. Eligible participants received outpatient primary care at pulmonary clinics of a single large US health system. Data were analyzed between 2021 and 2023.

**Exposure:**

Expectation accuracy, measured by comparing patients’ self-reported expectations of their symptom burden with their actual physical and emotional symptoms 3, 12, and 24 months in the future.

**Main Outcome and Measure:**

Health-related quality of life, measured by the St George’s Respiratory Questionnaire-COPD at 3, 12, and 24 months.

**Results:**

A total of 207 participants were included (median age, 65.5 years [range, 42.0-86.0 years]; 120 women [58.0%]; 118 Black [57.0%], 79 White [38.2%]). The consent rate among approached patients was 80.0%. Most patients reported no or only limited discussions of future health and symptom burdens with their clinicians. Across physical and emotional symptoms and all 3 time points, patients’ expectations were more optimistic than their experiences. There were no consistent patterns of measured demographic or behavioral characteristics associated with expectation accuracy. Regression models revealed that overoptimistic expectations of future burdens of dyspnea (linear regression estimate, 4.68; 95% CI, 2.68 to 6.68) and negative emotions (linear regression estimate, −3.04; 95% CI, −4.78 to 1.29) were associated with lower health-related quality of life at 3 months after adjustment for baseline health-related quality of life, forced expiratory volume over 1 second, and interval clinical events (*P* < .001 for both). Similar patterns were observed at 12 months (dyspnea: linear regression estimate, 2.41; 95% CI, 0.45 to 4.37) and 24 months (negative emotions: linear regression estimate, −2.39; 95% CI, −4.67 to 0.12; dyspnea: linear regression estimate, 3.21; 95% CI, 0.82 to 5.60), although there was no statistically significant association between expectation of negative emotions and quality of life at 12 months.

**Conclusions and Relevance:**

In this cohort study of patients with COPD, we found that patients are overoptimistic in their expectations about future negative symptom burdens, and such inaccuracies were independently associated with worse well-being over time. Developing and implementing strategies to improve patients’ symptom expectations may improve patient-centered outcomes.

## Key Points

**Question:**

Are patients’ individual health expectations associated with how they subsequently report health-related quality of life?

**Findings:**

In this cohort study of 207 outpatients with severe chronic obstructive pulmonary disease, most patients were overoptimistic about their future symptom burdens. Overoptimistic expectations of dyspnea burden and negative emotions were associated with worse health-related quality of life over a 24-month study period.

**Meaning:**

These results suggest that strategies to improve patients’ understanding of their illness and likely future symptom burden may improve decision-making and quality of life in serious illness care.

## Teaser

This cohort study compares what patients with severe chronic obstructive pulmonary disease expect to experience at diagnosis with reported experiences over a 24-month period.

## Introduction

Preference-sensitive, patient-centered health care supports individualized care that maximizes patients’ well-being.^1^ This inherently relies on patients forming expectations of future health and outcomes and choosing a course of action aligned with their values and those trajectories. Yet patients with serious illnesses may struggle to form accurate expectations. Doing so necessitates confronting the possibilities of increasing illness burden or death.^2,3,4,5,6^ Barriers to forming accurate expectations include tendencies to display present bias (ie, to overvalue the present),^7,8^ difficulty imagining unfamiliar health states,^9^ lack of coping skills, and preferences for receiving positive information.^10,11,12^ Without necessary support, patients with serious illnesses are likely to inaccurately anticipate their future health or avoid information about possible poor health outcomes.^13^

Prior work demonstrates that forming overoptimistic expectations may help some patients and caregivers cope with unfavorable prognoses, but also prevents adaptation to future hardships^14^ and impairs selection of behaviors or treatments most likely to result in desired outcomes.^2,3,15^ However, key knowledge gaps remain regarding the frequency and degree of inaccurate expectations, the directions of these inaccuracies, and the factors associated with expectation accuracy.

To inform the design of strategies to improve patients’ well-being and ensure the provision of patient-centered care, we sought to address these questions in the context of chronic obstructive pulmonary disease (COPD). COPD is the third-leading cause of death worldwide^16,17,18^ and causes significant dyspnea, functional limitations, social isolation, dependence on caregivers, and mood disorders.^19,20,21,22,23,24^ Despite this, patients with COPD infrequently engage in supportive and palliative care or enroll in hospice^25^ and have a high burden of hospitalizations including critical care.^25,26,27,28,29^ Patients with COPD in particular are theorized to be at risk for expectation inaccuracies,^9^ yet little is known about how patients perceive their future health in the context of this complex serious illness. We conducted a prospective cohort study measuring the accuracy of health expectations among patients with COPD. We hypothesized that many patients would hold overoptimistic expectations and that such expectations would be associated with decreased health-related quality of life (HRQL) based on our conceptual model. Some results have been previously reported as abstracts.^30,31^

## Methods

We conducted a prospective cohort study from December 2017 to July 2021. The institutional review board of the University of Pennsylvania approved the study protocol. We followed Strengthening the Reporting of Observational Studies in Epidemiology (STROBE) reporting guidelines for cohort studies. We recruited patients from primary care and pulmonary specialty outpatient clinics within a single, large health system. Potentially eligible patients were adults presenting for a scheduled appointment with a documented COPD diagnosis and at least 1 additional marker of severe illness.^32^ These included the use of long-term oxygen therapy, use of noninvasive ventilation for chronic hypoventilation, forced expiratory volume over 1 second (FEV_1_) less than 30% expected, body mass index (calculated as weight in kilograms divided by height in meters squared) below 21, recent hospital admissions for acute exacerbations of COPD (ie, 2 in the prior 2 years or 1 in the preceding year), severe co-occurring chronic illness (eg, noncurable malignancy, right heart failure, congestive heart failure, diabetes with end-organ damage, or kidney failure with an estimated glomerular filtration rate below 40 mL/min), and age older than 69 years. We excluded patients not fluent in written or spoken English, lacking capacity to make health care decisions, or with a non–COPD-related anticipated mortality within 6 months. If a patient was eligible based on a severe co-occurring chronic illness, the anticipated survival still had to exceed 6 months. Trained research staff systematically screened clinic appointment lists and approached potentially eligible patients in person. All participants provided informed consent at the time of enrollment (either written or verbally with a waiver of written consent) then completed surveys in person, over the phone or online. Participants received US $40 at the time of enrollment plus $20 for each follow-up, and 2 study-branded gifts worth $5.

### Assessment of Expectation Accuracy

Patients expected the frequency and degree of experiencing dyspnea using the Baseline Dyspnea Index (BDI) and Transition Dyspnea Index (TDI).^33,34^ The BDI captures dyspnea burden summed across 3 domains: functional impairment, magnitude of task, and magnitude of effort. The BDI can be completed in 4 to 5 minutes and is reliable.^35^ The TDI measures change in dyspnea from this baseline in the same domains.

At the time of enrollment, patients reported their dyspnea over the prior week using the BDI and made projections for 3, 12, and 24 months later using the TDI (eFigure 1 in Supplement 1). At each follow up, patients reported their actual symptoms also using the TDI. Therefore, each patient had 2 scores for each follow-up: the expected TDI score (ie, documented at the time of enrollment) and the actual TDI score. The accuracy of an individual patient’s expectations was determined by calculating differences between these 2 TDI scores at each time point (ie, the difference between the score they projected for 3 months later and their actual score at 3 months).

Using an instrument that assesses emotional expectations accuracy, each patient also reported their baseline and projected experience of 10 emotions^36^ using a 7-point Likert scale (eFigure 2 in Supplement 1). We calculated composite difference scores for positive and negative emotions separately.

Patients’ expectations for their survival may strongly affect their medical choices and health behaviors. At baseline, participants indicated how long they hope they will live,^1,2,9^ how long they expect they will live,^3^ and how long they believe their physicians estimate they will live (eFigure 3 in Supplement 1). Participants selected from categories for each: full life expectancy, more than 24 months but less than full life expectancy, 12 to 24 months, 3 to 12 months, or less than 3 months. Providing categories facilitated completion given the challenge of prognostic estimates^2^ and patients’ potential discomfort declaring exact survival estimates.^37^ We determined accuracy of lifespan expectations based on electronic health records (EHR) and, if needed, online obituary review to identify the date of death and compared this with their expected lifespan using the patients’ responses.

### Well-Being Outcome Variables

We assessed patient HRQL using the St. George’s Respiratory Questionnaire-COPD (SGRQ-C),^38,39,40^ a validated and widely used 40-item scale. The questionnaire measures how COPD affects overall health, daily life, and perceived well-being and is responsive to changes in individual circumstances.^38,40^ We assessed patients’ psychological distress as a secondary outcome, measured as depressive symptoms on the Patient Health Questionnaire-9 (PHQ-9).^41,42,43^ The PHQ-9 is a self-report questionnaire that is highly sensitive and specific for identification of major depressive disorder.^44,45^

### Other Measures

At baseline, we collected the site of enrollment, the presence of documented do not resuscitate or do not intubate orders, and treatments prescribed for COPD (eg, inhaled or oral medications, pulmonary rehabilitation, oxygen) from the EHR. Patients self-reported discussions with clinicians regarding expectations in emotional experiences, dyspnea, and survival, rating the depth of any discussions using a 5-point Likert scale. At each follow up, we collected interval use of pulmonary rehabilitation and new COPD treatments, unexpected (ie, not for a planned procedure) hospitalizations or COPD exacerbations, and newly diagnosed co-occurring conditions.

To advance knowledge of the mechanisms underlying well-documented disparities in the diagnosis, treatment, and outcomes of COPD,^46,47,48,49,50,51,52^ participants self-reported sociodemographic information including age, gender, ethnicity, race, level of education, household composition, employment status, household income, and tobacco use (historical and current). Because spiritual and religious beliefs may influence patients’ abilities and desires to form expectations^9^ and contribute to treatment decisions, adaptation, and stress reactions,^53,54^ participants completed the Systems of Belief Inventory (SBI-15R).^54^ We measured trait optimism, which may influence how patients cope with serious illness including their accuracy of expectations using the Life Orientation Test-Revised (LOT-R).^55,56,57,58^ This instrument operationalizes optimism, has good predictive validity, and has been used extensively.^59^ Finally, we measured temporal (or delay) discounting—ie, the decline in the value of a fixed reward when it is offered further in the future^60,61^—using a 9-item monetary choice questionnaire (MCQ) in which the patient chooses between larger financial rewards in the future or smaller rewards immediately.

### Statistical Analysis

We conducted descriptive statistics of the study sample, including sociodemographics, clinical characteristics, and expectation accuracies for each follow up time point. We then used classification and regression tree (CART) models to explore patient characteristics associated with expectation inaccuracies.^62^ CARTs are machine learning models, which identify a decision tree that describes patient expectations for different levels of an outcome. This nonparametric approach captures the association between the measured characteristics and separate observations into subgroups with similar outcomes. We chose this method over others because its output is easily interpretable and CART methods maintain good predictive accuracy. We handled missing baseline covariate data using a multiple imputation procedure, where the final CART hyperparameter was chosen as the average of the hyperparameters selected by cross-validation in each imputed data set.^63^ All CART models were fit using R statistical computing software version 4.1.2 (R Project for Statistical Computing) with the rpart package version 4.1.16.

To evaluate associations between patient’s expectation accuracy and future well-being, we generated multiple linear regression models. In the primary analysis, the exposure was patients’ difference scores between their 3-month expected and actual symptom burdens (emotions and dyspnea) and the outcome was HRQL at 3 months as measured by the SGRQ-C score. In secondary analyses, exposures included symptom projection accuracy at 12 and 24 months and survival projection accuracy at 3, 12, and 24 months, with outcomes being the SGRQ-C scores and PHQ-9 scores at 3, 12, or 24 months, as appropriate for the exposure. We included participants with baseline data and at least 1 follow-up in our analyses. Covariates included patients’ baseline SGRQ or PHQ-9 score, FEV_1_ at enrollment, interval pulmonary rehabilitation, interval hospitalizations, interval COPD exacerbations, and interval chronic illness diagnoses in the models as covariates likely to affect HRQL over time.

We estimated that enrolling 188 patients in the cohort would provide 80% power to detect a difference in SGRQ-C scores of 4 points between patients classified as optimistic as compared with those having accurate or pessimistic expectations at the 3-month follow-up, assuming approximately 75% would be optimists and a standard deviation of up to 16 points. The target effect size of 4 points is based on prior studies establishing this as the clinically meaningful change in SGRQ-C score.^38,40^ To account for estimated attrition of 10% by 3 months, we enrolled 207 patients in the cohort. We estimated that this sample size would provide greater than 80% power to detect a difference in difference scores of 2 points or more between patients who express inaccurate vs accurate understanding of treatment goals at the time of enrollment, which exceeds the minimum clinically significant change in TDI score.^64^ This power calculation assumed at least 75% of patients will hold an inaccurate understanding of treatment goals,^65^ a conservative standard deviation of 3 points,^36^ and an α = .05. We anticipated this sample size would allow for sufficient power to detect associations of continuous variables with expectation inaccuracy.

## Results

We enrolled 207 patients with an initial consent rate of 80.0% (median age, 65.0 years [range, 42.0-86.0 years]; 120 female [58.0%]; 118 Black [57.0%], 79 White [38.2%]) (Table 1). A total of 68 patients (56.1%) completed high school (secondary) or less formal education. At the time of enrollment, 55 (26.6%) reported active tobacco use. During the 24-month study period, 24.6% died or became too ill to participate, 15.0% withdrew or were lost to follow up for at least 1 time point, and 90.5% of living and medically able participants completed the study. In total, 69.1% of enrolled patients remained active in the study through 24 months (eFigure 4 in Supplement 1).

**Table 1.** Patient Characteristics at Baseline

Characteristic	Patients, No. (%) (n = 207)
Age, y	
Mean (SD)	65.5 (7.92)
Median (range)	65.0 (42.0-86.0)
Gender	
Male	85 (41.1)
Female	120 (58.0)
Nonbinary or genderqueer	2 (1.0)
Race	
Black	118 (57.0)
White	79 (38.2)
Other, multiracial, or choose not to answer^a^	10 (4.8)
Education	
12th grade or less (secondary)	68 (32.9)
Graduated from high school (secondary)	48 (23.2)
Some college (tertiary)	46 (22.2)
Graduated from college (tertiary)	26 (12.6)
Graduate school	19 (9.2)
Forced expiratory volume in 1 s, %	
Mean (SD)	51.5 (21.3)
Median (range)	50.0 (14.0-118)
Data not available, No. (%)^b^	8 (3.9)
St George’s Respiratory Questionnaire-COPD	
Mean (SD)	53.6 (21.0)
Median (range)	54.9 (0-97.5)
Data missing, No. (%)	23 (11.1)
Patient Health Questionnaire-9 score	
Mean (SD)	7.53 (5.92)
Median (range)	7.00 (0-23.0)
Data missing, No. (%)	2 (1.0)
Tobacco use (current)	
Every Day	29 (14.0)
Some Days	26 (12.6)
Not At All	135 (65.2)
Prefer not to answer	17 (8.2)
Systems of Belief Inventory Beliefs subscale	
Mean (SD)	24.9 (7.6)
Median (range)	28.0 (0-30.0)
Data missing, No. (%)	2 (1.0)
Systems of Belief Inventory Support subscale	
Mean (SD)	9.79 (5.0)
Median (range)	11.0 (0-15.0)
Data missing, No. (%)	3 (1.4)
Life Orientation Test-Revised	
Mean (SD)	16.7 (4.8)
Median (range)	17.0 (2.0-24.0)
Data missing, No. (%)	1 (0.5)
Monetary Choice Questionnaire Discount Rate (geometric mean)	
Mean (SD)	0.0780 (0.10)
Median (range)	0.0187 (0.000158-0.2)
Data missing, No. (%)	8 (3.9)
Extent patient discussed emotions with physician	
Very great extent	23 (11.1)
Great extent	14 (6.8)
Some extent	35 (16.9)
Little extent	12 (5.8)
Very little extent	8 (3.9)
No discussion	115 (55.6)
Extent patient discussed physical well-being with physician	
Very great extent	33 (15.9)
Great extent	41 (19.8)
Some extent	51 (24.6)
Little extent	5 (2.4)
Very little extent	8 (3.9)
No discussion	69 (33.3)
Extent patient discussed goals for medical care with physician	
Very great extent	29 (14.0)
Great extent	38 (18.4)
Some extent	40 (19.3)
Little extent	13 (6.3)
Very little extent	6 (2.9)
No discussion	81 (39.1)
Extent patient discussed future lifespan with physician	
Very great extent	10 (4.8)
Great extent	9 (4.3)
Some extent	11 (5.3)
Little extent	4 (1.9)
Very little extent	2 (1.0)
No discussion	170 (82.1)
Missing	1 (0.5)
Do not resuscitate or do not intubate order present	
Order	7 (3.4)
No order	200 (96.6)
Pulmonary rehabilitation (prior to baseline)	
No	122 (58.9)
Yes	85 (41.1)
Supplemental oxygen prescription	
No	95 (45.9)
Yes	112 (54.1)

Abbreviation: COPD, chronic obstructive pulmonary disease.

^a^ One participant self-identified as Hispanic and one as Native American; the remainder did not specify their race(s).

^b^ All patients had a clinical diagnosis of COPD in the electronic health record. For the purposes of this study, COPD could also be confirmed by findings of emphysematous changes on cross-sectional imaging.

The patients’ median FEV_1_ was 50.0% of expected with a median SGRQ of 54.9 (range, 0-97.5), indicating significant impairment of HRQL. At baseline, 170 patients (82.1%) reported that they had never discussed their expected lifespan with their physicians (Table 1), 115 patients (55.6%) reported they had never discussed their expected emotions, and 69 (33.3%) had never discussed their expected physical well-being. While most patients had discussed their goals of medical care with physicians, 81 (39.1%) reported no such discussions. During the 24-month study, 22 patients (10.6%) were overoptimistic in their survival projections with only 4 patients (1.9%) outliving their overly pessimistic survival projection.

Patients were overoptimistic across all 3 domains and all time points (Table 2; eFigure 5 in Supplement 1). For positive emotions, patients scored a mean (SD) 0.26 (1.23) points lower than expected at 3 months (95% CI, 0.09 to 0.44; *P* = .003), 0.49 (1.34) points lower than expected at 12 months (95% CI, 0.28 to 0.69; *P* = .01), and 0.53 (1.42) points lower at 24 months (95% CI, 0.30 to 0.77; *P* = .04). Patients scored their negative emotions a mean (SD) 0.19 (1.03) points higher at 3 months than initially projected (95% CI, −0.34 to −0.04; *P* < .001), 0.28 (1.14) points higher at 12 months (95% CI, −0.46 to −0.11; *P* = .001), and 0.29 (1.39) points higher at 24 months (95% CI, −0.52 to −0.06; *P* < .001). Patients also experienced more dyspnea than expected, scoring a mean (SD) 0.15 (0.98) points higher than projected at 3 months (95% CI, 0.01 to 0.29; *P* < .001), 0.48 (1.11) points higher at 12 months (95% CI, 0.31 to 0.65; *P* = .01), and 0.19 (1.21) points higher at 24 months (95% CI, −0.01 to 0.39; *P* = .07). These differences reflect the accuracy of patients’ personal projections of their future health, with the *P* values corresponding to *t* tests using a null hypothesis that the projections were accurate. In all cases, the mean projection was more optimistic than what was eventually observed (with all results significant except for dyspnea at 24 months), with trends toward greater inaccuracies at 12 and 24 months than at 3 months (eFigure 5 in Supplement 1). Patients expected to have positive emotions, lower amounts of negative emotions, and less dyspnea than they later experienced. CART analyses revealed no consistent patient characteristics associated with inaccurate expectations (eFigure 6, eTable in Supplement 1).

**Table 2.** Accuracy of Patient Expectations for Emotional and Dyspnea Outcomes at 3, 12, and 24 Months

Measure	Time	Mean difference (SD) [95% CI]^a^	*P* value^b^
Positive emotions	3 mo	0.26 (1.23) [0.09 to 0.44]	.003
	12 mo	0.49 (1.34) [0.28 to 0.69]	.01
	24 mo	0.53 (1.42) [0.30 to 0.77]	.04
Negative emotions	3 mo	−0.19 (1.03) [−0.34 to −0.04]	<.001
	12 mo	−0.28 (1.14) [−0.46 to −0.11]	.001
	24 mo	−0.29 (1.39) [−0.52 to −0.06]	<.001
Dyspnea	3 mo	0.15 (0.98) [0.01 to 0.29]	<.001
	12 mo	0.48 (1.11) [0.31 to 0.65]	.01
	24 mo	0.19 (1.21) [−0.01 to 0.39]	.07

^a^ Measures correspond to differences in expected and observed emotions and dyspnea at each time point. In all cases, patient expectations were more optimistic than what was eventually observed, with trends toward greater inaccuracies at 12 and 24 months than at 3 months.

^b^ Corresponding to *t* tests under a null hypothesis that the average difference in the expected and observed measure at each time point was equal to 0, ie, whether patient projections were on average correct.

Our linear regression models demonstrate that patients who hold overoptimistic expectations of their burdens of both dyspnea (linear regression estimate, 4.68; 95% CI, 2.68 to 6.68) and of negative emotions (linear regression estimate, −3.04; 95% CI, −4.78 to 1.29) had overall lower HRQL over 2 years (Table 3), even after adjusting for baseline FEV_1_ and interval receipt of pulmonary rehabilitation, hospitalizations, COPD exacerbations, and new chronic illness diagnoses. Negative emotion inaccuracy was associated with reduced HRQL at 3 and 24 months and increased depressive symptoms at 3 and 12 months, also adjusting for these other clinical factors (Table 4). Patients’ positive emotion expectation accuracy was not related to HRQL or depressive symptom burden at any time point.

**Table 3.** Associations Between Expectation Accuracy and Health-Related Quality of Life Over Time

Measure	Linear regression estimate (95% CI)^a^		
	3 mo	12 mo	24 mo
Baseline health-related quality of life	0.78 (0.69 to 0.87)^b^	0.79 (0.69 to 0.89)^b^	0.76 (0.63 to 0.90)^b^
Positive emotion expectation accuracy	1.05 (−0.37 to 2.47)	0.52 (−1.02 to 2.06)	0.18 (−1.79 to 2.14)
Negative emotion expectation accuracy	−3.04 (−4.78 to 1.29)^b^	−1.28 (−2.99 to 0.44)	−2.39 (−4.67 to 0.12)^c^
Dyspnea expectation accuracy	4.68 (2.68 to 6.68)^b^	2.41 (0.45 to 4.37)^c^	3.21 (0.82 to 5.60)^d^

^a^ Adjusted for baseline fractional expiratory volume over 1 second and interval receipt of pulmonary rehabilitation, hospitalization, exacerbation, and new chronic illness diagnoses.

^b^ *P* < .001.

^c^ *P* < .05.

^d^ *P* < .01.

**Table 4.** Associations Between Expectation Accuracy and Depressive Symptoms Over Time

Measure	Linear regression estimate (95% CI)^a^		
	3 mo	12 mo	24 mo
Baseline PHQ-9	1.22 (1.15-1.28)^b^	1.18 (1.11-1.26)^b^	1.25 (1.17-1.35)^b^
Positive emotion expectation accuracy	1.17 (0.91-1.51)	1.15 (0.87-1.53)	1.10 (0.82-1.47)
Negative emotion expectation accuracy	0.73 (0.55-0.98)^c^	0.70 (0.51-0.96)^c^	0.84 (0.61-1.17)
Dyspnea expectation accuracy	1.00 (0.72-1.39)	1.11 (0.77-1.59)	1.27 (0.91-1.78)

Abbreviation: PHQ-9, Patient Health Questionnaire-9.

^a^ Adjusted for baseline fractional expiratory volume over 1 second and interval receipt of pulmonary rehabilitation, hospitalization, exacerbation, and new chronic illness diagnoses.

^b^ *P* <.001.

^c^ *P* <.05.

## Discussion

This study suggests that unrealistically optimistic expectations of future health are associated with patients’ well-being in the context of serious, chronic illness. Most patients with COPD formed overoptimistic expectations, yet such expectation inaccuracies regarding future negative symptom burdens (ie, for dyspnea and negative emotions) were associated with lower HRQL over a 2-year period. These findings demonstrate that unrealized positive thinking may not support patients’ well-being over time. The lack of consistent patterns of sociodemographics associated with expectation accuracy suggests that individuals’ illness expectations are unlikely to explain differences or disparities in COPD care and outcomes.

Future-oriented thinking, or also referred to as “prospection,” is an essential foundation for decision-making.^66^ For health-related decisions, patients reflect on and form an understanding of the reversibility of their illness, risk of mortality, likely future symptom burdens, and the efficacy and potential adverse effects of available therapies. We found that most patients hold inaccurate expectations, inherently limiting their ability to make high-quality and value-aligned decisions. Health worsening unexpectedly as illness progresses is distinct from worse health that was anticipated. That is, one patient may hold accurate expectations about progression of a serious illness, while a different patient who experiences the same illness progression may hold overoptimistic expectations. In this way, their health progressed similarly but their expectation accuracy was different, likely influencing their coping and decision-making processes prior to and during this health decline.

The association of expectations on patient-centered outcomes provides a basis for developing novel interventions to complement other work promoting patient-centered decision-making. Most prior studies in this area have compared patient expectations with population rather than individual outcomes or focused on discrete interventions, such as measuring expectations before and after receiving a specific therapy.^3,65,67,68,69^ A strength of our study is that we focused on a debilitating and incurable chronic illness with difficult to predict illness trajectories, and we followed patients longitudinally, enabling calculation of patients’ actual expectation accuracy. This provides us critical longitudinal information about the accuracy with which patients think about their future health.

A second finding is that most patients had limited conversations with their clinicians about future symptom burdens and prognosis. Emotions, a dominant symptom in COPD,^70,71,72^ were particularly neglected as a topic of discussion. Prior work demonstrates that clinicians may fail to communicate negative prognoses in what they believe could be a form of benevolent deception.^73^ Given our findings, strategies to improve clinician communication and provide structured opportunities for patients to reflect on potential futures are likely to better align patients’ expectations with probable or potential outcomes. In this manner, clinician- or patient-facing interventions that improve expectation accuracy may directly improve patients’ serious illness experiences, alignment of medical decisions with their values, and clinical outcomes. Tools that provide patient-centered, tailored coping support may also result in a reduction of expectation inaccuracies.

### Strengths and Limitations

Strengths of this study include the novel use of longitudinal methods to measure expectation accuracy, excellent recruitment and retention, and inclusion of patient groups underrepresented in research. This study also had several limitations. First, our single health system sample did not represent rural populations disproportionately burdened by COPD and patients with limited English proficiency. Cultural differences across health systems or geographic regions could limit the generalizability of our findings.

Second, we did not measure all possible relevant behavioral characteristics. We prioritized the measurement of characteristics based on review of prior studies, evidence, and theoretical models. Finally, we only measured patients’ expectations without attention to family members and informal caregivers who may influence expectation formation, decisions, and outcomes.^74^ We are currently conducting additional work that builds from our findings and overcomes some of these limitations.

## Conclusions

Increased attention to prospection, or expectation formation, in chronic illnesses like COPD may enable patients to better prioritize and align their decisions with likely health trajectories. Patients with accurate expectations may be more able to engage in productive coping strategies. In this way, improving patients’ understanding of their illness trajectory may improve patients’ tendencies to make value-aligned medical decisions through more robust evaluation of the potential options and likely outcomes.^75^ Interventions, including coping support and communication tools targeted at patients and clinicians, must overcome the behavioral and psychological barriers to engaging in prospection when faced with a chronic illness.^66^ Therefore, our work complements and adds to the efforts to improve patient-centered, value-aligned decision-making and care planning in serious illness.

Our novel methods revealed that patients’ optimistic inaccuracies about negative symptom domains were associated with worse well-being over time. Despite this, patients report that health expectations are rarely discussed, even in the context of an incurable and debilitating illness. Developing and implementing tools and strategies to improve patients’ understanding of their illness and likely future symptom burdens, particularly those that lead to increased and effective patient-clinician communication, may improve patient-centered outcomes in serious illness care.

## Notes

Supplement 1. **eFigure 1.** Physical Symptom (Dyspnea) Example Item **eFigure 2.** Emotion Example Item **eFigure 3.** Life Expectancy Items **eFigure 4.** Participant Participation During the Longitudinal Study **eFigure 5.** Distribution of 3 Types of Symptom Accuracy at 3 Follow-up Time Points **eFigure 6.** Decision Tree Output from CART Models for Emotions and Dyspnea at 3 Months **eTable.** Evaluation of Emotion and Dyspnea Decision Trees (CART Models)

Supplement 2.
Data Sharing Statement
