# Supplementary material for: Accuracy of Expected Symptoms and Subsequent Quality of Life Measures Among Adults With COPD
Source: JAMA Netw Open. 2023 Nov 21;6(11):e2344030. doi: 10.1001/jamanetworkopen.2023.44030 (PMC10663971; doi:10.1001/jamanetworkopen.2023.44030)
Supplement: Supplement 1. — eFigure 1. Physical Symptom (Dyspnea) Example Item eFigure 2. Emotion Example Item eFigure 3. Life Expectancy Items eFigure 4. Participant Participation During the Longitudinal Study eFigure 5. Distribution of 3 Types of Symptom Accuracy at 3 Follow-up Time Points eFigure 6. Decision Tree Output from CART Models for Emotions and Dyspnea at 3 Months eTable. Evaluation of Emotion and Dyspnea Decision Trees (CART Models) [file jamanetwopen-e2344030-s001.pdf]

Supplemental Online Content

Hart JL, Summer AE, Ogunduyile L, et al. Accuracy of predicted symptoms and subsequent quality of life measures among adults with chronic obstructive pulmonary disease. *JAMA Netw Open.* 2023;6(11):e2344030.
doi:10.1001/jamanetworkopen.2023.44030

eFigure 1. Physical Symptom (Dyspnea) Example Item

eFigure 2. Emotion Example Item

eFigure 3. Life Expectancy Items

eFigure 4. Participant Participation During the Longitudinal Study

eFigure 5. Distribution of 3 Types of Symptom Accuracy at 3 Follow-up Time Points

eFigure 6. Decision Tree Output from CART Models for Emotions and Dyspnea at 3 Months

eTable 1. Evaluation of Emotion and Dyspnea Decision Trees (CART Models)

This supplemental material has been provided by the authors to give readers additional information about their work.

© 2023 Hart JL et al. *JAMA Network Open.*

eFigure 1. Physical Symptom (Dyspnea) Example Item

Prediction (baseline):

The following questions will ask you how you think your COPD symptoms will change in the future. For each item, imaging yourself in the future. Please select a single answer that describes how you expect to feel on a typical day 3 months, 1 year, and 2 years from now.

In 3 months, I expect that I will experience these changes in the activities that I do and how I feel:

- ◯ I will do much less so that I can avoid feeling short of breath. Activities will take me much longer to complete.
- ◯ I will avoid some activities in order to avoid feeling short of breath. I will have to stop and pause more when doing some activities.
- ◯ I will take my time more and do things more slowly to avoid feeling short of breath but I will not need to stop and pause more.
- ◯ There will be no change in how I do things.
- ◯ I will be able to do things more quickly without feeling short of breath.
- ◯ I will be able to do things with fewer breaks because I won't feel short of breath as easily.
- ◯ I will be able to do things with few, if any pauses. I will be able to do things much more quickly without feeling short of breath.

Actual symptom measurement (follow-up)

[3/12/24 months] ago, you described your shortness of breath symptoms as [baseline symptoms of dyspnea reported by the patient using the Baseline Dyspnea Index corresponding item].

Thinking about how things may have changed between that and now, please select the option that best describes your condition:

- ◯ I expend much less effort on tasks and activities so that I can avoid feeling short of breath. Activities now take me much longer to complete.
- ◯ I expend less effort in my activities in order to avoid shortness of breath. I need to pause more with some activities.
- ◯ I don't need to pause more to avoid shortness of breath, but I avoid activities that require a lot of effort so that I do not become breathless.
- ◯ There has been no change in effort to avoid shortness of breath.
- ◯ I am able to do things and exert more effort without shortness of breath. I sometimes carry out tasks more quickly than before.
- ◯ I am able to do things that require more effort with fewer pauses and without shortness of breath.
- ◯ I am able to do things that require much more effort with few or no pauses. I am able to do things much more quickly without feeling short of breath.

© 2023 Hart JL et al. *JAMA Network Open.*

eFigure 2. Emotion Example Item

Prediction (baseline):

I expect that I will feel sad

		Very Much			Somewhat			Not at all
1)	In 3 months	○	○	○	○	○	○	○
2)	In 1 year	○	○	○	○	○	○	○
3)	In 2 years	○	○	○	○	○	○	○

Actual symptom measurement (follow-up, including all emotional symptom words):

The following questions will ask you about the emotions that you experience.

For each item, please select a single answer that describes how you feel on a typical day during the past week.

	Very Much			Somewhat			Not At All
134) Sad	○	○	○	○	○	○	○
135) Happy	○	○	○	○	○	○	○
136) Angry	○	○	○	○	○	○	○
137) Nervous	○	○	○	○	○	○	○
138) Enthusiastic	○	○	○	○	○	○	○
139) Jittery	○	○	○	○	○	○	○
140) Hostile	○	○	○	○	○	○	○
141) Excited	○	○	○	○	○	○	○
142) Lonely	○	○	○	○	○	○	○
143) Content	○	○	○	○	○	○	○

© 2023 Hart JL et al. *JAMA Network Open.*

eFigure 3. Life Expectancy Items

In this section, you'll answer questions about your expectations regarding your life span.

The following questions will ask you to think about how long you expect that you have remaining in your life. For each of the questions, please select a single answer that you think is the most accurate.

1)	Knowing that you have a serious illness, how long do you hope to live?	◯ A full life expectancy<br>◯ More than 2 years from now but less than a full life expectancy<br>◯ More than 12 months from now but less than 2 years from now<br>◯ Less than 12 months from now<br>◯ I don't know<br>◯ I choose not to answer
2)	Knowing that you have a serious illness, how long do you think that you have to live? (This may be the same or different from how long you hope to live)	◯ A full life expectancy<br>◯ More than 2 years from now but less than a full life expectancy<br>◯ More than 12 months from now but less than 2 years from now<br>◯ Less than 12 months from now<br>◯ I don't know<br>◯ I choose not to answer
3)	Knowing that you have a serious illness, how long does your physician think that you have to live?	◯ A full life expectancy<br>◯ More than 2 years from now but less than a full life expectancy<br>◯ More than 12 months from now but less than 2 years from now<br>◯ Less than 12 months from now<br>◯ I don't know<br>◯ I choose not to answer

© 2023 Hart JL et al. *JAMA Network Open.*

eFigure 4. Participant Participation During the Longitudinal Study

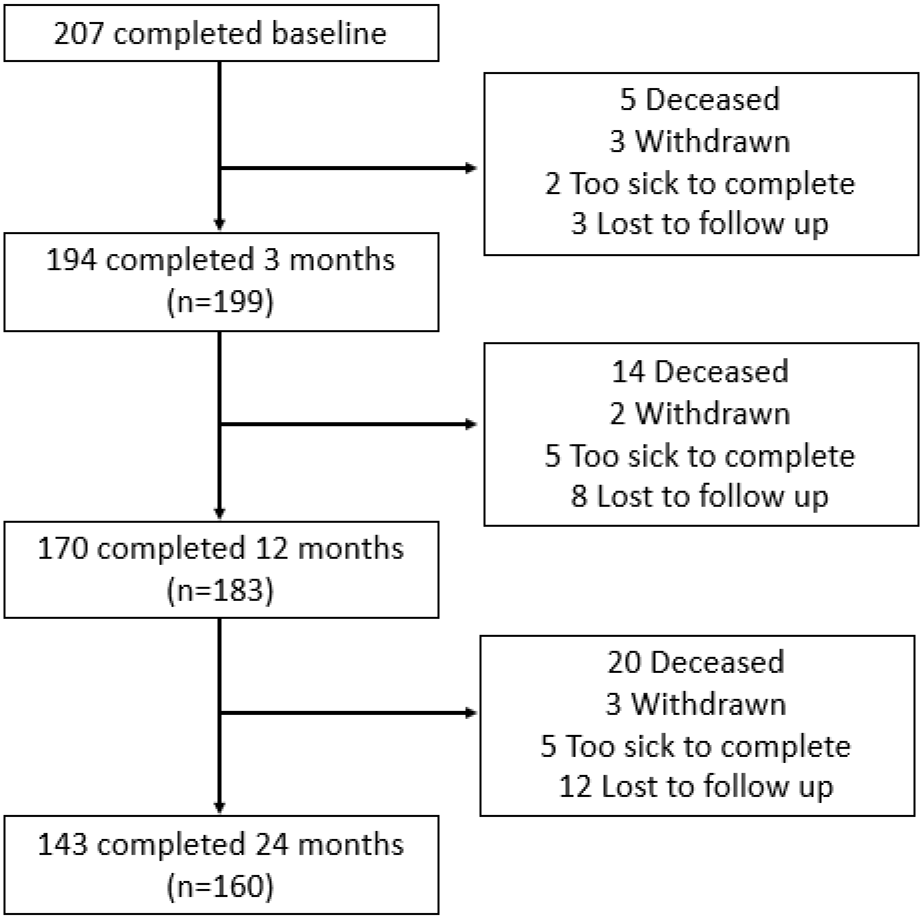

© 2023 Hart JL et al. *JAMA Network Open.*

eFigure 5. Distribution of 3 Types of Symptom Accuracy at 3 Follow-up Time Points

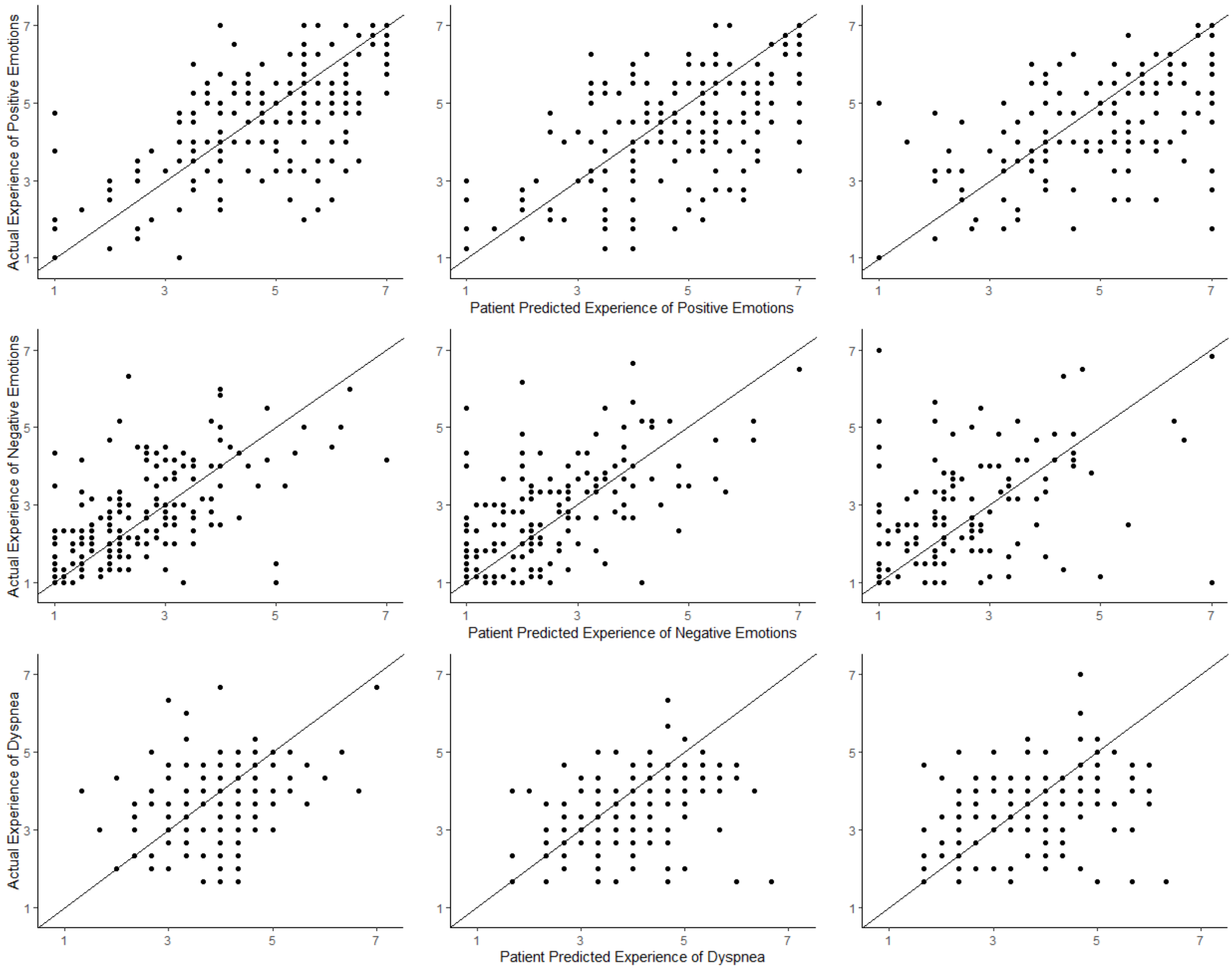

We plotted each patient's predicted versus actual symptom burdens for each of the three symptom domains and each of the three follow-up time points. The *difference score* used in our analysis is the distance between the plot and the center line. This center line demonstrates where patients holding accurate predictions (i.e., if the patient's predicted and the actual symptom burden was the same) would be plotted.

© 2023 Hart JL et al. *JAMA Network Open.*

eFigure 6. Decision Tree Output from CART Models for Emotions and Dyspnea at 3 Months

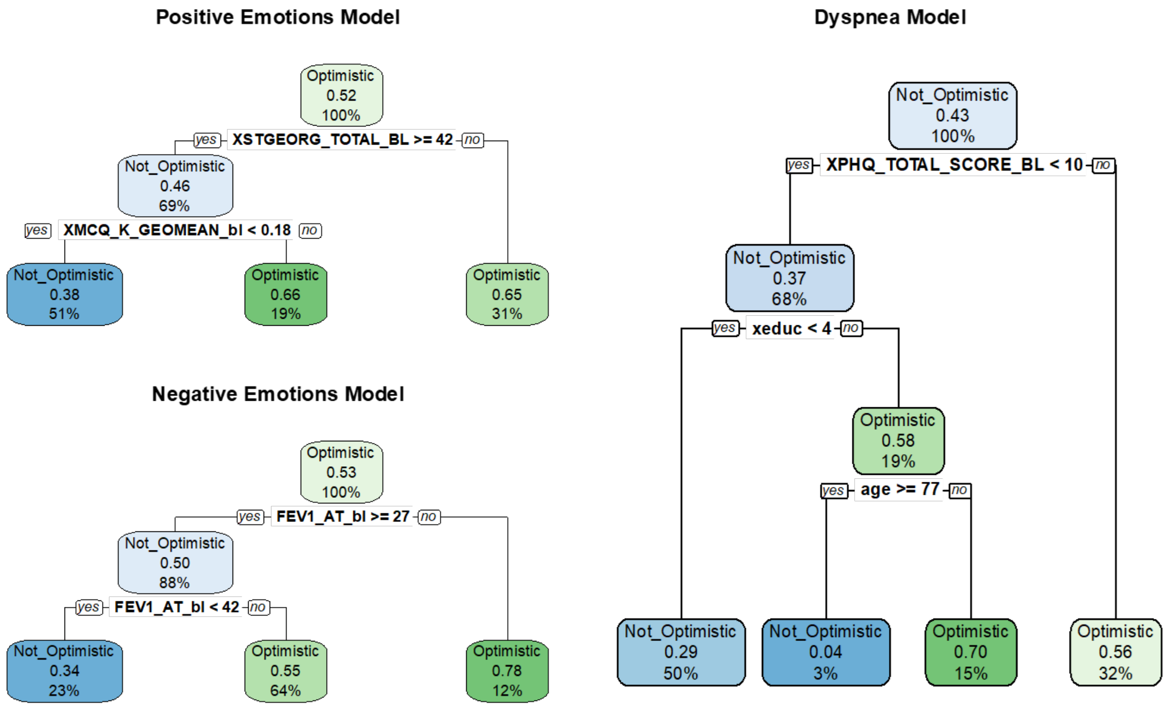

<u>Classification and regression model (CART) results</u>

CARTs are machine learning models which identify a decision tree that predicts different levels of an outcome (here, whether or not patients had optimistic expectations). This non-parametric approach captures the relationships between the measured characteristics to separate observations into subgroups with similar outcomes. Missing baseline covariate data were handled using a multiple imputation procedure, where the final CART hyperparameter was chosen as the average of the hyperparameters selected by cross-validation in each imputed data set.[84] All CART models were fit using R statistical computing software v. 4.1.2[85] with the 'rpart' package v. 4.1.16.

In our CART analyses, we identified that individuals with clinically significant depressive symptoms (PHQ-9 score ≥10) and individuals with PHQ-9 scores <10 but who were younger than 77 years old and college graduates were overly optimistic on average in their predictions for dyspnea at 3 months. All other individuals are expected to make pessimistic (or neutral) predictions. We also found that individuals with depressive symptoms (PHQ-9 scores ≥6) and less temporal discounting (MCQ scores <0.18) at baseline made pessimistic/neutral predictions for positive emotions, while all others tended to be overly optimistic in their predictions. Finally, patients with FEV1 between 27% and 42% predicted at baseline tended to make pessimistic/neutral predictions for negative emotions, while those with higher or lower FEV1 at baseline tended to make optimistic predictions for negative emotions. There was no predictive role for SBI-15R, LOT-R, or other measured characteristics. Model accuracy for each of these decision trees is described in eTable 1.

© 2023 Hart JL et al. *JAMA Network Open.*

© 2023 Hart JL et al. *JAMA Network Open.*

eTable 1. Evaluation of Emotion and Dyspnea Decision Trees (CART Models)

Metric	Positive Emotions	Negative Emotions	Dyspnea
Specificity	0.71	0.85	0.68
Sensitivity	0.53	0.33	0.67
NPV	0.63	0.66	0.73
PPV	0.63	0.58	0.61

PPV = Positive Predictive Value; NPV = Negative Predictive Value; the optimistic category was treated as “positive”

© 2023 Hart JL et al. *JAMA Network Open.*
