# Supplementary material for: Accuracy of Expected Symptoms and Subsequent Quality of Life Measures Among Adults With COPD
Source: JAMA Netw Open. 2023 Nov 21;6(11):e2344030. doi: 10.1001/jamanetworkopen.2023.44030 (PMC10663971; doi:10.1001/jamanetworkopen.2023.44030)
Supplement: Supplement 2. — Data Sharing Statement [file jamanetwopen-e2344030-s002.pdf]

Data Sharing Statement

Data

Data available: No

Additional Information

Explanation for why data not available: We will work with individual investigators requesting a copy of the data to ensure compliance with the IRB-approved study protocol given the limitations on data sharing included in the consent provided by patient participants. Please email joanna.hart@pennmedicine.edu to coordinate data sharing.
